# Cellular telephone use during free-living walking significantly reduces average walking speed

**DOI:** 10.1186/s13104-016-2001-y

**Published:** 2016-03-31

**Authors:** Jacob E. Barkley, Andrew Lepp

**Affiliations:** College of Education, Health and Human Services, Kent State University, White Hall, Kent, OH 44242-000 USA

**Keywords:** Mobile phones, Physical activity, Walking, Exercise

## Abstract

**Background:**

Cellular telephone (cell phone) use decreases walking speed in controlled laboratory experiments and there is an inverse relationship between free-living walking speed and heart failure risk. The purpose of this study was to examine the impact of cell phone use on walking speed in a free-living environment.

**Methods:**

Subjects (*n* = 1142) were randomly observed walking on a 50 m University campus walkway. The time it took each subject to walk 50 m was recorded and subjects were coded into categories: cell phone held to the ear (*talking, n* = 95), holding and looking at the cell phone (*texting, n* = 118), not visibly using the cell phone (*no use, n* = 929).

**Results:**

Subjects took significantly (*p* < 0.001) longer traversing the walkway when *talking* (39.3 s) and *texting* (37.9 s) versus *no use* (35.3 s).

**Conclusion:**

As was the case with the previous laboratory experiments, cell phone use significantly reduces average speed during free-living walking.

## Background

Presently 98 % of young adults own a cellular/mobile telephone (cell phone) and they use these devices heavily (>4 h day^−1^) [1]. There is also evidence that this heavy cell phone use occurs in seemingly every imaginable setting (e.g., in college classes, while driving, in bed, during sex, in the shower etc.) [2, 3]. In other words, many young adults are using their cell phones often and everywhere. Because of the omnipresence of cell phone use, researchers have begun to examine how these devices may be affecting other behaviors and behavioral outcomes. For example, excessive cell phone use has been linked with an increased incidence of traffic accidents, elevated anxiety, lack of sleep, poor academic performance and lower cardiorespiratory fitness [2, 4–12]. Taken together these findings indicate that excessive cell phone use may be considered a negative health behavior.

Previously, our group has examined associations between cell phone use and a range of health behaviors and outcomes [5, 13, 14]. We have reported an inverse relationship between cell phone use and cardiorespiratory fitness and a positive relationship between cell phone use and sedentary behavior [5, 13]. Additionally, we examined the effect of cell phone use on average walking speed during a bout of treadmill exercise in a controlled laboratory environment [14]. Using the cell phone to talk or send text messages significantly decreased treadmill walking speed relative to a condition with no cell phone use. This result is similar to another laboratory study by Parr et al. that utilized motion capture and an 8 m track to demonstrate that subjects had impaired gait mechanics and walked more slowly while texting versus a condition with no cell use [15]. These findings are potentially important if they translate to actual walking behavior as walking is the most commonly reported form of physical activity [16, 17].

There is evidence that individuals use their cell phones while walking and that this use is associated with increased risk of injury [7–11]. However, other than the aforementioned laboratory-based studies, there is only a single study that we are aware of which examined how cell phone use affects free-living walking pace in a field setting [18]. This previous study examined the effects of cell phone use on attention while walking across a large open area on a college campus. The open area was a central student gathering place and posed a “complex navigational task” (p. 599) for the subjects. It demonstrated that, under these conditions, talking on a cell phone while walking led to inattentiveness, increased weaving and directional change, and greater time needed to traverse the open area relative to non-users. However, it remains unknown if cell phone use increased the time it took to complete this “complex navigational task” because cell use actually slowed walking pace or if it simply added to the difficulty of traversing the open area (e.g., increased weaving and directional change). If the greater time to traverse the open area was due to increased weaving and direction change and not a decrease in walking speed, cell phone use while walking may actually increase total walking behavior while maintaining speed. This would be a positive outcome. To assess whether cell phone use actually decreases free-living walking speed there is a need to investigate how cell phone use affects free-living walking in a setting relatively free of distraction and navigational challenge. In addition, this prior study only examined talking on a cell phone. There is a need to assess the effect of increasingly common cell-phone based activities that require the user to look at the device (e.g., texting, watching a video).

A greater understanding of how cell phone use may affect walking behavior is important as walking is the most commonly reported form of physical activity and for many individuals walking for active transport (i.e., getting from one place to another) is the only daily physical activity they participate in [16, 17]. While the amount of walking (i.e., duration, distance) individuals participate in is positively associated with a number of health benefits, walking pace is emerging as a stronger predictor of cardiovascular disease risk. Recent articles from Boone-Heinonen et al. and Saevereid et al. both indicated that walking pace, more strongly than distance or duration, was inversely associated with cardiovascular disease risk factors and congestive heart failure [19, 20]. Therefore, if individuals regularly use their cell phones while walking and this use reduces walking speed then it is possible that these slower walking speeds could increase the risk of cardiovascular disease.

The purpose of this study was to compare average speed during free-living, active-transport walking while individuals were holding a cell phone to their ear (*talking*), actively utilizing the phone with their hands and looking at the screen (*texting*), or not using a cell phone (*no use*). Naturalistic observations were made, unbeknownst to the subjects, in a field setting as individuals traversed a 50 m straight walkway on an American college campus. We hypothesized that individuals who were talking or texting on a cell phone would walk more slowly than individuals who were not using their cell phone.

## Methods

**Fig. 1:**
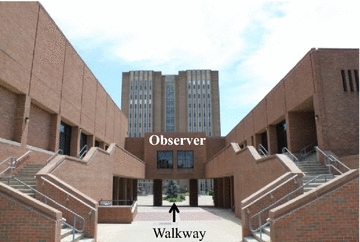
Photograph of the 50 m walkway and overpass from which research personnel observed the subjects

Research personnel randomly observed *N* = 1197 individuals traverse a 50 m portion of a walkway of a large, public university in the Midwestern United States. Observations were made by two graduate research assistants who were trained simultaneously by one of the co-principal investigators (co-PI, Lepp). The observational task was relatively simple in that there were only four variables to record per observation (sex, seconds to traverse path, type of cell phone use, whether or not the person being observed was wearing headphones), the course was clearly marked and the observation post (Fig. 1) provided an excellent vantage point. Additionally, after training the observers, the co-PI stayed at the site for a minimum of 30 min and practiced the observational technique with each of two observers until the co-PI was confident the observations between the two graduate research assistants agreed with his. Observations were made between the months of April and August of the same year and only on days where there was no rain or threat of rain. Temperature ranged from 48° to 86 ℉. Observations were made on each day of the week, and at different times of day (during daylight hours only), in an effort to obtain a representative sample. Research personnel observed subjects while looking through a window of an overpass that bisected the 50 m walkway from above (Fig. 1). This allowed for the naturalistic observations to be made by research personnel without the knowledge of the subjects walking below who remained anonymous throughout the entirety of the study. The walkway was straight, had limited ingress and egress, and was simple to navigate. All observations were made while subjects were walking in one direction (west to east) approaching the overpass where research personnel were positioned. The starting and end points to the 50 m walkway were selected as they had physical landmarks (e.g., a park bench bolted to the walkway) that were easily seen by the observer. Walking speed was recorded, via a stopwatch, as the time (seconds) it took subjects to walk from the start to end points on the 50 m walkway. Walking speed was recorded for every fifth individual, walking independent of others (i.e., alone), to cross the starting point on the 50 m walkway. Subjects were coded into the following four, separate categories:

1. *Talking* subject held a cell phone to their ear for the entirety of the time it took them to traverse the 50 m walkway (*n* = 95).
2. *Texting* subject held a cell phone in their hands and was looking at the screen for the entire 50 m walkway (*n* = 118).
3. *Partial use* subject participated in one of the two aforementioned cell phone activities for a portion, but not the entirety, of the 50 m walkway (*n* = 51).
4. *No use* subject did not exhibit any visible aforementioned use (e.g., *talking* or *texting*) of a cell phone for the entire 50 m walkway (*n* = 929).

Subjects were also coded as male or female and whether or not they were wearing headphones. These factors were recorded to be used as co-variates as sex is a well-established correlate to physical activity behavior and listening to music has been shown to increase walking speed [21–24]. Subjects who fell into the *partial use* category were removed from all subsequent data analysis leaving 1142 subjects categorized into three groups for comparison: *talking, texting* and *no use*.

While the proportion of the total sample that was using their cell phone for the entire 50 m walkway (18.7 %) was smaller than those not, the total number of observations (*n* = 213) was more than four times greater than the number of observations (*n* = 43) made by Hyman et al. of individuals walking while using their cell phones [18]. Furthermore, to achieve a power of ≥0.80 and an α ≤ 0.05 for the comparison of time to traverse the 50 m path during *talking* (39.3 s) and *texting* (37.9 s) versus the no use category (35.3 s), *n* = 63 (*texting*) and 26 (*talking*) subjects would be needed, respectively. Therefore the current samples of 95 (*talking*) and 118 (*texting*) were deemed to be adequate.

All methods were approved by the University Institutional Review Board (IRB) at Kent State University. Because this was a naturalistic observation study and subjects were unaware that they were being observed the need for informed consent was waived by the IRB.

### Data analysis

All data were analyzed using the Statistical Package for the Social Sciences (SPSS version 21). A three cell phone use category (*talking, texting, no use*) univariate analysis of co-variance (ANCOVA), co-varying for sex (male, female) and headphone use group (headphones on, no headphones), was utilized to assess differences in the time it took subjects to traverse the 50 m walkway. Post-hoc mean comparisons with the Bonferroni adjustment were used to examine any significant effects from the ANCOVA.

## Results

In this snapshot of free-living walking, 18.7 % of the study subjects were actively utilizing their cell phone (*talking* or *texting*) for the entire 50 m portion of the walkway observed for this study. Univariate ANCOVA revealed a significant [*F*(2, 1141) = 31.7, *p* < 0.001, power = 1.00] main effect of cell phone use category for walking time, independent of sex and headphone use group. Post-hoc comparisons with the Bonferroni adjustment revealed that subjects in both the *talking* (39.3 ± 5.4 s) and *texting* (37.9 ± 5.7 s) categories took a significantly (*p* < 0.001) greater amount of time traversing the walkway (i.e., walked more slowly) versus those in the *no use* (35.3 ± 4.8 s) category. There was no difference (*p* = 0.13) between the *talking* and *texting* categories. There was also a significant [*F*(1, 1141) = 30.9, *p* < 0.001, power = 1.00] main effect of sex as males (34.9 ± 5.0 s, *n* = 555) traversed the walkway more quickly than females (36.8 ± 5.0 s, *n* = 587). There was no difference [*F*(1, 1141) = 2.3, *p* = 0.13, power = 0.33] in walking speed in participants wearing headphones (35.3 ± 5.1 s, *n* = 276) versus those not wearing headphones (36.1 ± 5.1 s, *n* = 867).

## Discussion

This is the first study that we are aware of to examine the effect of cell phone based *talking* and *texting* (or similar behavior) on active-transport walking behavior in a natural setting relatively free of distraction and navigational challenge [18]. Presently, relative to subjects who were not using a cell phone, subjects walked 11.3 and 7.4 % more slowly while *talking* and *texting*, respectively. These results were independent of sex and whether or not subjects were using headphones and are in support of the prior field-based study and experimental studies from our group and others that examined the effect of cell phone use on walking behavior in controlled laboratory environments. Hyman et al. noted that participants talking on cell phones took 10.3 % longer to traverse a large, complex courtyard than non-cell phone users [18]. Hyman et al. noted a greater amount of time needed to traverse a “complex navigational path” (10.3 %) while talking on a cell phone which was very similar to the reduced walking speed noted in the present study (11.3 %) and a reduction noted (10.7 %) in our own previous laboratory studies [14]. All of these studies examined walking pace while participants were talking on a cell phone. Parr et al. utilized an optical motion capture system to examine the effect of cell phone texting on walking speed and mechanics across an 8 m track [15]. Relative to a condition with no cell phone use participants exhibited several negative changes in gate mechanics and an associated 8.5 % reduction in walking speed. Again, this is very similar to the reduction in walking speed while texting noted in the present study (7.4 %) and reductions we previously reported (10.7 %) in participants texting while walking on a treadmill [14]. Taken together, the present field-based findings and previous field and laboratory-based findings provide support for the notion that cell phone use (*talking* and *texting*) may decrease walking speed whether it is planned exercise (i.e., treadmill walking) or active transport (i.e., free-living walking). This intensity-dampening effect of cell phone use on physical activity may serve as a mechanism explaining a previously demonstrated inverse relationship between cell phone use and cardiorespiratory fitness [5].

Walking is the most common form of physical activity that individuals report participating in [16, 17]. As such, its health benefits have been well studied [25–28]. While the amount (duration, distance) of walking has repeatedly been shown to have positive effects on cardiovascular disease risk (CVD), a thorough review article by Boone-Heinonen et al. noted that walking pace (speed) had a particularly robust inverse relationship with CVD risk [19]. Similarly, Saevereid et al. examined the associations between the development of heart failure and daily walking duration and speed [20]. They noted that while the duration of walking was inversely associated with the risk of heart failure in women, this was not the case in men. Furthermore, when adjusting for confounding factors (e.g., age, family history smoking status, etc.), this relationship was no longer present for women either. Conversely, there was a dose–response inverse relationship between walking speed and the risk of heart failure for both men and women. The relationship remained significant even after adjusting for confounding factors. This evidence indicates that average daily walking speed may be of greater importance than duration in the prevention of CVD. If this is the case, the reduced walking speeds noted among cell phone users in the present study coupled with the heavy use of cell phones in nearly all daily activities is concerning [1, 3]. If individuals use their cell phones often while walking and this use reduces walking speed as we have demonstrated, this reduced walking speed could increase the risk of CVD over time.

This study is not without limitations. First, while this research was conducted at a large public university (enrollment >28,000 students), it is possible that some of the 1197 observations may have included the same individual multiple times. Second, while we feel that observing unaware individuals is a strength of this study, we were only able to make these observations in a limited space (i.e., 50 m path). Because this is a relatively short distance it may not be an ideal representation of daily walking behavior. Third, because the study is non-experimental we cannot infer causality. While we note reduced walking speed with cell phone use we cannot know if the cell phone use caused this lower speed or if individuals that walk more slowly for other reasons are more likely to use their cell phones than faster walking peers. Future studies may consider observing an individuals’ free-living walking behavior before and after they initiate cell phone use to examine fluctuations in speed within those individuals. However, in previous laboratory-based examinations of the effect of cell phone use on walking behavior there were significant reductions noted in walking speed during conditions where the participant was required to use their phone for talking and/or texting versus a condition with no cell phone use [14, 15]. Therefore, while we cannot infer causality in the present study, the similarity between the current results and those in the previous, laboratory-based studies would suggest that cell phone use is likely to blame for the lower walking speeds.

In conclusion, this was the second study we are aware of that demonstrated reduced free-living, active-transport walking speed during cell phone *talking* and the first to do so during cell phone *texting* versus no cell phone use. These results also support the findings of previous laboratory-based evidence that demonstrated that cell phone use reduced walking speed. This suppressed walking speed is worrisome as average walking speed has been shown to be a strong negative predictor of CVD risk. While more research is needed we would advise against cell phone use during free-living, active-transport walking as it may diminish the health benefits of this activity.
